# Rapid loss of antipredatory behaviour in captive-bred birds is linked to current avian invasions

**DOI:** 10.1038/srep18274

**Published:** 2015-12-15

**Authors:** Martina Carrete, José L. Tella

**Affiliations:** 1 Department of Physical, Chemical and Natural Systems, Universidad Pablo de Olavide, Sevilla, Spain; 2 Department of Conservation Biology, Estación Biológica de Doñana, Consejo Superior de Investigaciones Científicas (CSIC), Sevilla, Spain

## Abstract

Despite the importance of behaviour in conservation biology, there have been few studies that address behaviour in areas such as invasion ecology. There is an urgent need to identify specific traits that facilitate the establishment and spread of alien species to prevent biological invasions and their impact on biodiversity. Changes in antipredatory behaviour in captivity have been proposed to explain the higher invasiveness of wild-caught exotic species. We experimentally tested this hypothesis by assessing the response of wild-caught and captive-bred cage birds facing an approaching predator and their ability to escape from human capture, using species available in the Spanish pet market. Results showed the loss of antipredatory responses and escape abilities in captive-bred birds compared with wild-caught ones. An intraspecific comparison between wild-caught and the first generation of captive-bred birds pointed to a rapid behavioural loss in captivity (individual lifetime) rather than to differences among species (evolutionary exposure). In the context of current avian invasions, the proportion of individuals showing antipredatory responses within a species was positively related to the likelihood of the species being found escaped and breeding in the wild. These results offer a link between behaviour, fitness, and the invasion syndrome in birds.

Exotic species represent one of the major threats to native biodiversity and the correct functioning of ecosystems, with worldwide social and economic implications^1^,^2^. Eradication programs can be extremely costly^3^ and ineffective^4^. By the time such programs are implemented, invasive species may have already caused long-term changes in the ecosystem^5^. Therefore, preventing invasions by recognizing potential invaders early on is proposed as the most effective management option. Specific traits that facilitate the establishment and spread of alien species are often difficult to identify, and ecologists have mainly investigated life history traits and ecological requirements of species to predict their invasiveness^6^,^7^. Previous studies have identified behavioural flexibility as a major determinant of invasion success of deliberately introduced birds^8^. However, the question of whether temperaments (also termed personalities, behavioural syndromes, or coping styles^9^,^10^,^11^) could be important factors in the invasiveness syndrome^12^,^13^,^14^ remains open.

Contrary to past deliberate introductions, recent (and current) avian invasions mainly arise from the accidental escape of globally traded cage birds^15^,^16^,^17^,^18^,^19^. In this relatively novel scenario, Carrete & Tella^15^ showed that wild-caught cage bird species are much more prone to escaping and becoming invaders than those bred in captivity, regardless of their availability on the pet market. The ability to cope with new environments could have been lost in species bred in captivity over a long period of time, while the high mortality rates during the international trade of wild-caught birds could have selected individuals with life-history and physiological traits that positively influence the establishment success of these groups of species^20^. Indeed, wild-caught and captive-bred individuals differ in their responses to acute stress. Wild-caught individuals show longer CORT responses to acute stress than captive-bred ones, both at inter- and intra-specific levels^21^. The longer acute response found in wild-caught birds could help them escape from cages and survive better when facing challenges in new environments, possibly contributing to their higher invasiveness.

Another potential explanation for the higher invasiveness of wild-caught species is the maintenance of antipredatory behaviours, important components of coping in the wild, which could be lost in species bred in captivity^14^,^19^. Predation is considered one of the most important selective pressures on free-ranging animals. Thus, any individual whose behaviour facilitates the evasion of predators or escape when attacked will have a greater probability of surviving to breed and, therefore, a greater probability of producing offspring^22^,^23^. However, some behavioural responses to predators are costly -they must be traded off with other activities such as feeding, resting or looking for mates^24^. Thus, they are plastic^25^,^26^ and lost when prey face low predation pressure^27^ or are isolated from predators, such as happens on islands^28^ or when animals are brought into captivity^29^. Independent of the way in which antipredator behaviour is modified when animals are isolated from predators^30^, these behavioural changes may have important consequences in conservation biology and the management of endangered species. Training procedures to avoid predators have been implemented as a component of captive rearing and translocation programs aimed to restore populations of threatened species^24^,^31^. However, less attention has been paid when considering potential links with other conservation problems such as biological invasions^32^.

Here, we experimentally test the hypothesis that wild-caught and captive-bred individuals differ in their antipredatory responses using a large sample of the exotic cage bird species most commonly traded and maintained in captivity (i.e., parrots and songbirds)^15^,^33^. Our inter- and intraspecific approaches show that the antipredatory behaviours as well as the ability to elude capture by humans are rapidly lost in captive-bred birds compared to wild-caught ones, just in the first generation of captive breeding. Moreover, we found a significant link between loss of antipredatory behaviour and invasiveness of the experimentally tested species, suggesting a relevant role during the latter stages (introduction, establishment and spread) of the invasion process.

## Results

### Antipredatory behaviour

**Figure 1:**
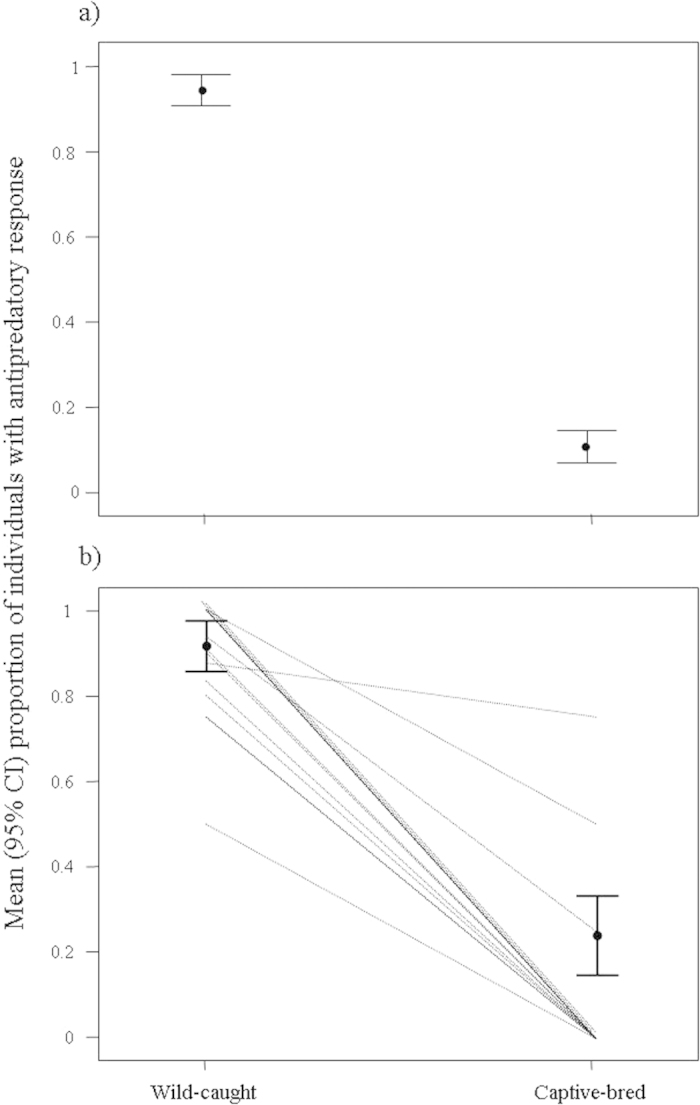
(**a**) Interspecific approach: mean proportion of individuals from wild-caught (n = 39) and captive-bred (n = 52) species showing antipredatory responses; (**b**) Intraspecific approach: mean proportion of wild-caught and captive-bred conspecific individuals with antipredatory response (n = 16 species). Dashed lines represent the species-specific changes in mean antipredatory behaviours. 95% CI for means are shown in both figures.

We experimentally assessed the antipredatory behaviour in 422 individuals (157 wild-caught and 265 captive-bred birds) belonging to 93 exotic species (57 species of parrots and 36 species of passerines) available in the pet market. In a first interspecific approach, 94.3% of individuals (n = 157) from wild-caught species (n = 39) showed antipredatory responses, while only 10.9% of individuals (n = 210) from captive-bred species (n = 52) responded to the predator model. Wild-caught species were more prone to eliciting antipredator responses when facing a predator than captive-bred ones (Fig. 1a, χ^2^ = 215.38, df = 1, p < 0.0001), while controlling for body size (χ^2^ = 0.58, df = 1, p = 0.45) and taxonomic effects.

Due to the difficulty of obtaining first generation birds born in captivity from wild-caught parents, sample sizes were reduced to 140 individuals from 12 species of parrots and 4 species of passerines when we evaluated intraspecific changes in antipredatory behaviour (S1). Most of the wild-caught individuals (90%, n = 81) responded to the predator, contrasting with the low percentage (10%, n = 50) among the conspecifics born in captivity. It is worth noting that changes in the response to the predator related to captivity were nearly identical when comparing this intraspecific approach (Fig. 1b) to the interspecific one (Fig. 1a). The antipredator response was significantly reduced in captive-born individuals compared to wild-caught conspecifics (Fig. 1b, χ^2^ = 92.91, p < 0.0001), supporting the hypothesis that the loss of this behaviour is related to a bird’s origin but not to interspecific differences.

Of the wild-caught individuals introduced into the predation-risk simulator and faced with a plastic object similar in size and colour to the predator (16 passerines from 8 species; S1), none showed antipredatory responses. This contrasts with the 94.6% of conspecifics of the same origin (wild-caught) that were exposed to the stuffed predator model (Yates’ corrected χ^2^ = 37.28, p < 0.001, n = 55). This, together with the differential responses of wild-caught and captive-bred conspecifics to the predator model, allowed us to be confident that the above results were indicative of antipredatory behaviour instead of fear of an approaching object.

### Escape abilities

**Figure 2:**
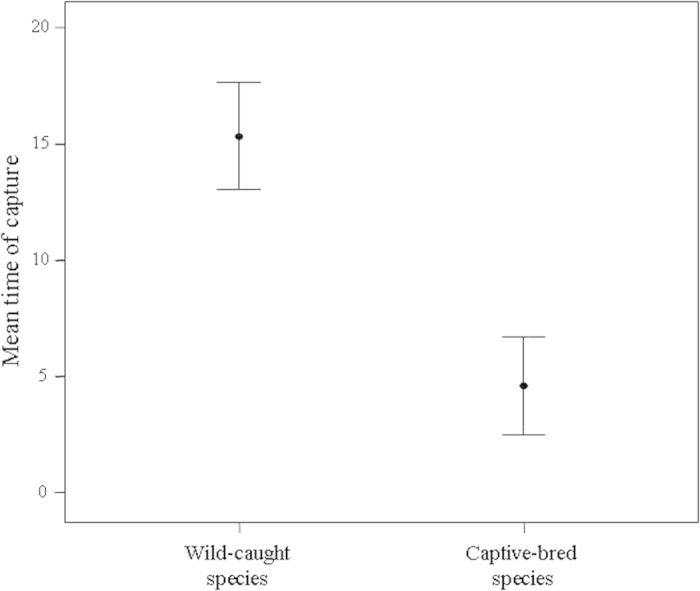
Mean time (in seconds) needed to capture by hand wild-caught (n = 26) and captive-bred (n = 14) passerine species. 95% CI for means are shown.

Capture time was measured for 228 individuals belonging to 40 species of passerines (S2). The time taken to capture by hand was shorter for captive-bred birds than for wild-caught birds (F_1,35_ = 93.82, p < 0.001). As shown in Fig. 2, capture time for wild-caught individuals was more than three times that required to capture captive-bred ones.

### Field evidence

After accidentally escaping from cages, 30 out of 33 captive-bred birds were recaptured (17 by hand or using a handled net, 4 by a dog, 9 came back voluntarily to the cage) while 21 out of 23 wild-caught birds eluded recapture. The percentage of recaptured captive-bred birds (90.91%) was significantly higher than that for wild-caught ones (8.70%, Yate’s χ^2^ = 34.13, p < 0.0001, n = 56). These percentages were nearly the inverse to the percentages of captive-bred and wild-caught birds experimentally responding to the predator in both the inter- and intraspecific approaches (see above), and thus inferences from our experimental approach are supported by observations of the outcome of actual escapes from cages.

### Antipredatory behaviour as a predictor of invasiveness

**Figure 3:**
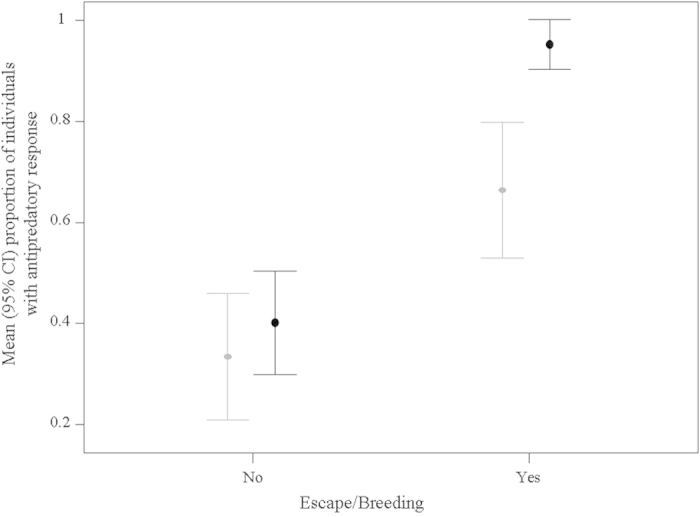
Proportion of individuals showing antipredatory behaviour related to whether or not the species were observed in the wild after escaping from cages (grey symbols) or were observed breeding in the wild (black symbols).

Escaped species showed a higher proportion of individuals responding to the stuffed predator than non-escaped species (χ^2^ = 9.46, p = 0.0021; Fig. 3). Among escaped species, those that established breeding populations in the wild showed a higher percentage of individuals with antidepredatory behavior (χ^2^ = 15.36, p < 0.001; Fig. 3). These results are not affected by temporal trends in the escape of wild-caught and captive-bred species (Authors, in prep). The percentage of individuals with antipredatory behaviour explained a low percentage of deviance in escape likelihood (7.65%), but this percentage increased for breeding likelihood (26.52%), supporting the importance of this behaviour for coping in the wild and establishing breeding populations.

## Discussion

Understanding how and under what circumstances individuals survive agonistic encounters, mainly predation, may help us to understand the selective forces involved in the transition of species through the last stages of the invasion process (introduction, establishment and spread^34^). In this sense, our results are clear in showing the loss of antipredatory behaviours as well as escaping abilities in birds bred in captivity compared with wild-caught ones. This behavioural change occurs very quickly, as suggested by our intraspecific comparison between wild-caught individuals and the first generation of captive-bred birds, which were not in contact with predators, supporting the idea that antipredatory behaviours develop within an individual’s lifetime^27^. Predator avoidance acquired through social learning is widespread across taxa (i.e., fish, birds, eutherians, and marsupials^35^). The general pattern is that before learning, individuals show little or no response toward predation stimulus, but after that stimulus has been presented together with an alarm signal or a true predation experience, it evokes an avoidance response^36^. Our results are in line with this pattern of antipredatory response acquisition: birds born in captivity, in the absence of predation risk, may not have learned their species-specific predatory behaviours when facing a predator from their wild parents or conspecifics. There is, however, a small proportion (ca. 10%) of captive-bred individuals, in both inter- and intraspecific comparisons, that responded to the predator approach. This may likely be due to the fact that some aviculturists breed exotic birds in outdoor facilities, where they are in contact with wildlife (including aerial predators) and thus do not lose their antipredator behaviours (authors' own observations and experience). We could not test this since the studied captive-bred individuals were bought in pet shops and so their conditions during captive-rearing were unknown.

Apart from conspecific transmission of antipredatory behaviour, it is well known that many birds have been selectively bred over generations for domestication, which may generate behavioural changes as a by-product^37^. Thus, changes in the antipredatory behaviour of captive-bred birds can be consequence of their isolation from predators but also due to founder effects and/or artificial (human) selection^25^. Both novel conditions seen in captivity and husbandry practices would have favoured particular geno/phenotypes^38^,^39^. For example, neophobic or highly active and explorative animals could be more likely to be poorly adapted to captive conditions than less active or explorative animals^40^,^41^. Moreover, individual temperament traits are often correlated, such that those individuals that are relatively bold towards predators are also more aggressive towards conspecifics (including mates and offspring; see references in^42^), thus reproducing worse in captivity. In this way, neophobic individuals with a bold and aggressive personality would be scarce in captivity, contributing to a reduction in variability in the full range of temperaments of captive-bred birds^25^,^33^ and thus, promoting a medium-term loss of antipredatory response at the population level.

Humans may deliberately attempt to modify animal temperaments, but human-induced changes are often unexpected and unplanned, with detrimental side effects of human activities like the captive breeding of wild animals^24^,^25^. Here, we have shown how a behavioural consequence of captive breeding usually viewed as detrimental for the success of conservation strategies such as reintroduction or translocation plans^43^,^44^ can be exploited to tackle a serious conservation problem like current exotic invasions. This offers a mechanism for explaining why wild-caught pet species are much more successful as invaders than captive-bred ones^13^. Moreover, the intraspecific comparison discards the possibility that captive species were long ago selected against others because of their adaptable temperament to captivity, since the antipredatory behaviour is rapidly lost after the selection of species for breeding (during an individual lifetime) without marked taxonomic differences (evolutionary time). This loss becomes an advantage when considering that pet birds born in captivity can be easily removed from nature by predators and humans after escaping from cages. On the contrary, the use of wild-caught birds as pets represents a potential risk for conservation of local biodiversity since, when escaped, they can establish viable populations and eventually became biological invaders^13^. Altogether, our results (mainly the relationship between the response toward a predator and the establishment success of exotic species) demonstrate the fitness implications of variability in antipredatory behaviour, as birds whose behaviour reduces the risk of predation are more likely to survive and breed^17^, and therefore to establish self-sustained populations in the wild.

Regarding management implications, it is difficult to control exotic bird populations once they are established^45^ and our results reinforce the polemic, controversial need for banning the trade of wild-caught birds as a preventive action (see review in^13^). A permanent trade ban (as enforced in Spain by law Real Decreto 630 in 2013) worldwide would be an easy and effective way of controlling current, and preventing future, exotic invasions, while captive breeding can satisfy the social demand for exotic cage birds without risks^1313^.

## Material and Methods

### Sampled birds

Wild-caught and captive-bred exotic birds of the Order Psittaciformes and Passeriformes, commonly used as cage birds^15^,^46^, were bought in pet markets in 2005 for our experimental purposes. Thus, they represented a random sample of individuals coming from different countries and/or aviculture breeders. Rather than testing antipredatory responses in many individuals from a few species, we chose to use a few individuals from many species and families to better represent the diverse availability of cage birds in the pet market^15^,^46^. The main origin (wild-caught or captive-bred) of cage bird species was easily determined at the time we sampled them in the Spanish pet markets. On the one hand, there were many species whose exportation of wild-caught individuals was banned long ago and thus, all individuals available in Spain originated from decades (e.g., all Australian species) and even centuries (e.g., the canary *Serinus canaria)* of breeding in captivity. On the other hand, all individuals available in the pet market from many other species were imported after being caught in the wild (mostly in Neotropical and African countries), as shown for Spain in the CITES trade database where the wild-caught or captive-bred origin of traded individuals is reported (www.cites.org). Individuals from these species were sold with accompanying CITES documentation that demonstrated their origin. Finally, there were some predominantly wild-caught species for which some captive-bred individuals were available in the pet market due to the recent efforts of aviculturists to breed them in captivity. We made an effort to sample some of those first-generation (F1) captive-bred individuals for our intraspecific approach. As an example, the monk parakeet (*Myiopsitta monachus*) was traded worldwide and imported by Spain by the thousands as wild-caught individuals^18^. While wild-caught was clearly the main origin for this species (see also www.cites.org), we were able to get a few individuals bred in captivity by aviculturists. Those captive-bred individuals were easily recognised as they are individually marked as chicks with un-removable closed bands with the year of birth and the code of the aviculturist engraved. Moreover, the price of captive-bred individuals was 2–3 times higher than that of their wild-caught conspecifics, reflecting their rarity in captivity. We classified these species with a mixed origin of individuals as predominantly wild-caught, since wild-caught individuals broadly represented >90 % of individuals available in the market. The origin of all sampled individuals and the main origin of all species sampled are shown in S1 and S2.

Attending to the individual CITES documentation, wild-caught birds were collected in the field at least one year before initiating our experiments and held in captivity until that time. Both captive-bred and wild-caught birds were at least 1 year old.

### Antipredatory behaviour

**Figure 4:**
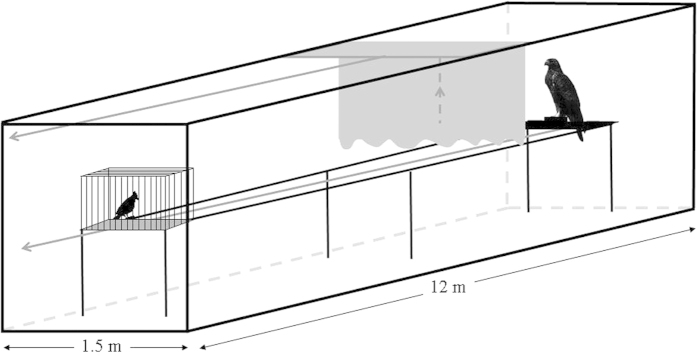
Predation-risk simulator used to evaluate the response of birds to the approach of a predator.

We assessed the response of birds in the presence of a predator by using a *predation-risk simulator* (Fig. 4). After keeping birds in cages (1 × 0.4 × 0.5 m) for two weeks for standardizing housing conditions, birds were individually placed into an identical cage located on one side of a tunnel, while at the other end we placed a taxidermic model of a medium-sized raptor (common buzzard *Buteo buteo*) hidden behind an opaque curtain (Fig. 4). We allowed birds to remain undisturbed in the experimental arena for 30 minutes. Then, we raised the curtain, and moved the raptor model towards the bird at a constant speed (0.5 m/s) through pulley mechanisms. The researcher observed through a small one-way window, thus being undetected by the focal bird and not interfering with its antipredatory behaviour. We considered that birds responded positively if they elicited some kind of antipredatory response such as escaping, freezing or alarm calling while the stuffed predator was approaching the cage. Otherwise, when birds continued without changing their normal behaviour until the stuffed predator was 50 cm from the cage, we considered that they did not respond to a potential predation event. To be confident that birds responded to the predator but not just to the approaching movement of an object, we confronted a subset of individuals not used in the experiment but belonging to the same species that elicited antipredatory responses (see results) to a plastic object similar in size and colour to the predator (a portable, brown water pump) as a control test. All tests were conducted on the earlier and later hours of the day, thus avoiding the central hours when birds reduce their activity.

Since differences in the antipredatory response towards mammals and avian predators have been previously reported^47^, we chose a raptor model because most predators of birds in Europe (and thus in Spain, where we focused our current avian invasion model^15^) belong to this group (31 species of diurnal raptors preying upon birds, of which 15 are specialised on birds). On the contrary, only two mammal carnivores predate regularly on birds at night (when birds are roosting, so opportunities for enemy recognition and selection of antipredatory behaviour are few), and their abundance and range distribution are very limited. Therefore, using a stuffed mammal predator in the experiments would add little to our conclusions on invasions.

### Escape abilities

Besides being caught by a predator, exotic birds that have escaped from cages are often recaptured by humans to be housed again as cage pets. Thus, we performed a second experiment using small passerines (<25 g) as study models to record time (in seconds) required to capture by hand focal individual birds in a standard cage of 0.5 × 0.4 × 0.5 m. This size allowed the researcher to easily access all corners of the cage, thus avoiding biases related to handling facilities. Parrots were not included in this experiment since their movements may have been restricted in this reduced space, and variability in aggressiveness and biting defence among species may have affected the willingness and ability of the researcher to capture them. All trials were performed by the same researcher (JLT).

### Field evidence

During a ten-year period (2005-2015), 47 out of ca. 1,200 exotic parrots and passerines escaped accidentally from the experimental facilities and aviaries, when some cages were accidentally opened or broken due to different causes. Moreover, nine free-living exotic birds were attracted to the aviaries by the captive birds. We tried to catch all escaped birds using a handled net, while some birds were killed by a small-sized dog in the surrounding garden before we had the opportunity to capture them. We used this information, from 18 species of parrots and 18 species of passerines, as additional evidence supporting the hypothesis of a greater ability to elude capture in wild-caught than in captive-bred pet species once escaped from cages.

### Antipredatory behaviour and invasiveness

We tested whether the antipredatory behaviour of the experimentally tested exotic species was linked to their invasiveness, using information on invasive exotic birds in Spain^48^ and complemented with a large data set that compiles >13,000 records of >75,000 individuals from > species of non–native bird species observed in the wild in Spain and Portugal up to the end of 2012^46^,^49^,^50^. We assessed the transitions of experimentally tested species through two main stages of the invasion pathway^34^, i.e. escape (introduction) and breeding (establishment) in the wild (S1). We considered as escaped species those that have been observed by ornithologists in the wild in Spain. We considered as breeding species the subset of escaped species that have been recorded breeding in the wild without human assistance, sometimes with self-sustainable, or even spreading, wild populations. We did not consider established and spreading species separately because of the relatively small sample size, the difficulties in determining whether species are established or spreading^37^, and the fact that many of the established species founded breeding populations very recently and will probably spread in the near future^46^. We excluded from the analysis of species breeding in the wild two whydahs (Family Viduidae, see S1) since their obligate brood parasitism may impair their invasive ability^15^.

### Data analyses

We used Generalized Linear Mixed Models (GLMM) to establish the effect of captivity on the probability of detecting differences in antipredatory responses between wild-caught and captive bred birds (link function: logistic, error distribution: binomial). Using sample size as denominator in the binomial GLMMs allowed us to satisfactorily compare prevalence data (i.e., the proportion of birds responding to the antipredatory trial) when sample sizes are usually small and variable among species^51^. First, in an interspecific approach we tested if wild-caught species showed a higher proportion of individuals with antipredatory responses than captive bred ones. In this way, we only considered the behaviour of individuals belonging to the main origin (i.e., wild-caught or captive bred) in the case of species for which both wild-caught and captive-bred individuals were sampled (S1). For example, we were able to sample 11 wild-caught and 11 captive-bred monk parakeets (S1), but only the 11 wild-caught individuals were included in this statistical test because wild-caught was the main origin for this species (see above). Potential differences in antipredatory responses linked to species’ size^52^ were controlled for by including their mean body mass (author’s unpublished data^53^) as a covariate in the analysis (body mass range: 8–357 g), while we controlled for potential taxonomic effects by including family and order as nested random factors in the GLMMs^8^. Second, for an intraspecific approach, we compared wild-caught individuals with captive-bred individuals born from wild-caught parents (F1). We aimed to disentangle whether differences in antipredatory responses were truly linked to the origin of individuals (wild-caught or captive bred) and thus acquired through the lifetime. Alternatively, differences could be linked to unmeasured specific traits or evolutionary exposure. We sought to evaluate how rapid the loss of antipredatory behaviour was. For example, in the case of the monk parakeet we compared the behaviour of the 11 wild-caught individuals with that of the 11 captive-bred ones (S1). We controlled for potential taxonomic effects by nesting species on family and order as a random factor in the model. Capture time (in seconds) of individuals from wild-caught and captive-bred passerine species were also compared through GLMMs (link function: log, error distribution: Poisson), also including species and family as nested random terms. In this case, there were no species including both wild-caught and captive-bred individuals (S2). Body mass was not controlled for in this analysis since all tested species were small-sized passerines (S2). Finally, we performed GLMM, controlling for taxonomic relationships as a random term, for assessing the likelihood of species to escape and breed in the wild (logit link function and a binomial error distribution) in relation to their antipredatory behaviour. We used as an explanatory variable the proportion of individuals that responded toward predators in the *predation-risk simulator* (see before) and included only those belonging to the main origin of each species (i.e., wild-caught or captive bred). For the example of the monk parakeet, only the 11 wild-caught individuals (S1) were used to calculate the proportion of individuals showing antipredatory behaviour.

### Ethics Statement

JLT held the Spanish certificates (B and C) that legally allow the design and conduct of experimental research work using live animals. Work in captivity was done under institutional approval of the competent Spanish wildlife agency (Consejeria de Medio Ambiente, Junta de Andalucía) and from the Ethic Committee of CSIC (CEBA-EBD-12-48), in the authorized centre for experimental avian research SE/16/U (REGA ES410910008016). Methods were carried out in accordance with the approved guidelines.

## Additional Information

**How to cite this article**: Carrete, M. and Tella, J. L. Rapid loss of antipredatory behaviour in captive-bred birds is linked to current avian invasions. *Sci. Rep*. **5**, 18274; doi: 10.1038/srep18274 (2015).

## Supplementary Material

Supplementary Information
