# Supplementary material for: Rapid loss of antipredatory behaviour in captive-bred birds is linked to current avian invasions
Source: Sci Rep. 2015 Dec 15;5:18274. doi: 10.1038/srep18274 (PMC4678868; doi:10.1038/srep18274)
Supplement: Supplementary Information [file srep18274-s1.pdf]

Rapid loss of antipredatory behaviour in captive-bred birds is linked to current avian invasions

Martina Carrete[1,2]* and José L. Tella[2]

[1]Department of Physical, Chemical and Natural Systems, Universidad Pablo de Olavide, Sevilla, Spain

[2]Department of Conservation Biology, Estación Biológica de Doñana, Consejo Superior de Investigaciones Científicas (CSIC), Sevilla, Spain.

Appendix 1. Species and number of individuals used in the experiment of antipredatory behaviour, including their origin (wild-caught or captive-bred), the number of wild birds used as experimental controls, and whether the species have been recorded escaped and breeding in the wild.

	Main origin of species	*Wild individuals*	*Captive individuals*	*Wild controls*	*Escaped*	*Breeding*
**Order Psittaciformes**						
**Family Psittacidae**						
Agapornis canus	Wild	16	4		Yes	No
Agapornis fischeri	Captive		4		Yes	No
Agapornis personatus	Captive		2		Yes	No
Alisterus scapularis	Captive		3		No	No
Amazona albifrons	Wild	4	2		Yes	No
Aprosmictus erythropterus	Captive		3		No	No
Ara (=Orthopsittaca)manilata	Wild	2			No	No
Ara (=Diopsittaca) nobilis	Wild	4	1		Yes	No
Aratinga (=Thectocercus) acuticaudata	Wild	4	1		Yes	Yes
Aratinga (=Eupsittula) aurea	Captive		4		Yes	No
Aratinga auricapillus	Captive		4		No	No
Aratinga jandaya	Captive		2		Yes	No
Aratinga (=Psittacara) leucophtalmus	Wild	2	2		Yes	No
Aratinga (=Psittacara)mitratus	Wild	1			Yes	Yes

Aratinga (=Eupsittula) pertinax	Wild	8	4	Yes	No
Aratinga solsticialis	Captive		2	No	No
Aratinga (= Pisttacara) wagleri	Wild	1		Yes	No
Barnadius zonarius	Captive		3	Yes	No
Bolborhynchus lineola	Captive		10	No	No
Brotogeris jugularis	Captive		2	No	No
Cyanoliseus patagonus	Wild	6	3	Yes	Yes
Cyanoramphus auriceps	Captive		3	No	No
Cyanoramphus novaezelandiae	Captive		9	No	No
Forpus coelestis	Captive		4	No	No
Forpus conspicillatus	Captive		6	No	No
Forpus passerinus	Captive		4	Yes	No
Lathamus discolor	Captive		5	Yes	No
Melopsittacus undulatus	Captive		6	Yes	No
Myiopsitta monachus	Wild	11	11	Yes	Yes
Nandayus (=Aratinga) nenday	Wild	10	2	Yes	Yes
Neophema (=Neopsephotus)bourkii	Captive		4	No	No
Neophema elegans	Captive		3	No	No
Neophema pulchella	Captive		7	Yes	No
Neophema splendida	Captive		1	No	No
Pionites melanocephalus	Wild	1	2	Yes	No
Pionus senilis	Wild	2	2	No	No
Platycercus elegans	Captive		4	Yes	No
Platycercus icterotis	Captive		4	Yes	No
Poicephalus senegalus	Wild	8		Yes	Yes

Polytelis alexandrae	Captive		2	No	No
Polytelis anthopeplus	Captive		3	No	No
Polytelis swainsonii	Captive		5	No	No
Psephotus dissimilis	Captive		4	No	No
Psephotus haematonotus	Captive		2	Yes	No
Psephotus varius	Captive		6	No	No
Psittacula cyanocephala	Captive		4	No	No
Psittacula eupatria	Captive		1	Yes	No
Psittacula finschii	Wild	1	1	No	No
Psittacula krameri	Wild	2	5	Yes	Yes
Pyrrhura cruentata	Captive		2	No	No
Pyrrhura egregia	Captive		2	No	No
Pyrrhura molinae	Captive		4	Yes	No
Pyrrhura perlata	Captive		2	No	No
Pyrrhura rhodocephala	Captive		2	No	No
Pyrrhura roseifrons	Captive		4	No	No
Pyrrhura rupicola	Captive		3	No	No
**Family Cacatuidae**					
Nymphicus hollandicus	Captive		8	Yes	No
**Order Passeriformes**					
**Family Sturnidae**					
Cinnyricinclus leucogaster	Wild	4		No	No
Lamprotornis chalybaeus	Wild	3		Yes	Yes

**Family Passeridae**						
Passer luteus	Wild	4		2	Yes	No
**Family Ploceidae**						
Euplectes afer	Wild	1			Yes	Yes
Euplectes franciscanus	Wild	5	1	1	Yes	No
Euplectes hordeaceus	Wild	3		1	Yes	No
Euplectes nigriventris	Wild	1			Yes	No
Ploceus cucullatus	Wild	4			Yes	Yes
Quelea quelea	Wild	5		1	Yes	Yes
**Family Estrildidae**						
Aidemosyne modesta	Captive		4		No	No
Amadina fasciata	Wild	7		2	Yes	No
Amandava amandava	Wild	2	4		Yes	Yes
Chloebia gouldiae	Captive		12		Yes	No
Emblema guttata	Captive		4		No	No
Erythrura psittacea	Captive		4		No	No
Estrilda astrild	Wild		4		Yes	Yes
Estrilda caerulescens	Wild	1			Yes	No
Lonchura bicolor	Wild	3		3	Yes	No
Lonchura cantans	Wild	5	2	3	Yes	No
Lonchura castaenothorax	Captive		4		No	No
Lonchura fringilloides	Wild	6		3	No	No

Lonchura malacca	Wild	1		Yes	Yes
Lonchura punctulata	Wild		2	Yes	Yes
Neochmia ruficauda	Captive		3	Yes	No
Padda oryzivora	Captive		4	Yes	No
Poephila acuticauda	Captive		7	Yes	No
Poephila bichenovii	Captive		6	Yes	No
Poephila personata	Captive		4	No	No
Pytilia hypogrammica	Wild	1		No	No
Uraeginthus bengalus	Wild	2		Yes	No
**Family Fringillidae**					
Carpodacus mexicanus	Captive		2	No	No
Serinus canaria	Captive		3	Yes	No
Serinus leucopygius	Wild	7	2	No	No
Serinus mozambicus	Wild	3		Yes	No
**Family Viduidae**					
Vidua chalybeata	Wild	4		Yes	
Vidua macroura	Wild	2		Yes	

Appendix 2. Species and number of individuals used in the experiment of escape abilities, including their origin (wild-caught or captive-bred).

	Wild	*Captive*
**Family Estrildidae**		
Aidemosyne modesta		10
Amandava amandava	4	
Amandava subflava	10	
Amadina fasciata	8	
Chloebia gouldiae		24
Emblema guttata		5
Erythrura psittacea		7
Erythrura trichroa		3
Estrilda astrild	5	
Estrilda caerulescens	1	
Estrilda melpoda	5	
Estrilda troglodytes	5	
Lagonosticta senegalus	1	
Lonchura bicolor	9	
Lonchura cantans	10	
Lonchura castaenotorax		4
Lonchura fringilloides	10	
Lonchura malaca	1	
Lonchura punctullata	2	
Neochmia ruficauda		13
Padda oryzivora		9

Poephila acuticauda		9
Poephila bichenovii		4
Poephila cincta		4
Poephila guttata		5
Poephila personata		2
Pytilia hypogrammica	1	
Uraeginthus bengalus	5	
**Family Ploceidae**		
Euplectes afer	4	
Euplectes franciscanus	7	
Euplectes hordeaceus	3	
Quelea erythrops	1	
Quelea quelea	5	
**Family Passeridae**		
Passer luteus	6	
**Family Fringillidae**		
Serinus canaria		9
Serinus leucopygius	5	
Serinus mozambicus	5	
**Family Emberizidae**		
Sicalis flaveola	1	

Family Viduidae

Vidua chalybeata	4
Vidua macroura	2
